# Accuracy of Clinical Suspicion for Rotator Cuff Tears by Orthopedic Surgeons When MRI Was Ordered on Initial Visits: Should Physical Therapy Be Mandated by Insurance Before MRI?

**DOI:** 10.7759/cureus.62079

**Published:** 2024-06-10

**Authors:** Caroline T Gutowski, Nicholas Pohl, Matthew Stern, Pietro M Gentile, Christopher Rivera-Pintado, Parker H Johnsen, Krystal Hunter, Catherine Fedorka

**Affiliations:** 1 Medicine, Cooper Medical School of Rowan University, Camden, USA; 2 Orthopaedic Surgery, Cooper University Health Care, Camden, USA; 3 Orthopaedic Surgery, Cooper University Hospital, Camden, USA; 4 Biostatistics, Cooper University Hospital, Camden, USA

**Keywords:** rotator cuff repair, healthcare costs, insurance, magnetic resonance imaging, physical therapy, atraumatic rotator cuff tears

## Abstract

Introduction: Insurance companies often mandate six weeks of physical therapy (PT) prior to approving MRIs for patients with atraumatic rotator cuff (RTC) tears. While this is designed to limit unnecessary imaging orders, it can increase healthcare costs and delay diagnosis and surgery. This study investigated the incidence of full- and partial-thickness tears when an MRI was ordered at the time of initial consultation for shoulder pain by an orthopedic provider.

Methods: A retrospective review of patients who had an MRI ordered upon initial orthopedic consultation for chronic shoulder pain was conducted. The primary outcome measured was the presence of RTC tears as determined by the MRI report. The cost of six weeks of PT versus the cost of immediate MRI in these patients was collected from our institution’s financial database. ANOVA, independent T-test, and chi-square test were used to analyze the differences between groups.

Results: A total of 365 patients were included. There were no significant differences in demographics between patients with full, partial, or no tears, with the exception that patients with full-thickness tears were older. Specifically, 43.0% had a full-thickness tear, 24.7% had a partial-thickness tear, and 32.2% had no tear on MRI. A total of 56.1% of the full-thickness tears proceeded to surgery. The cost of an upper extremity MRI without contrast averages $2,268, while two sessions of PT per week for six weeks totals $2,328.

Discussion: Over 67% of MRI orders yielded a positive finding of an RTC tear and remained at 67.2% in the absence of a history of conservative treatment, validating a specialist's clinical suspicion for an RTC tear and indication for MRI. Pre-MRI PT to satisfy insurance requirements may therefore delay intervention and increase healthcare costs when an orthopedic provider believes an MRI is warranted for clinical decision-making.

## Introduction

Rotator cuff (RTC) pathologies are responsible for nearly 4.5 million healthcare visits per year, with tears affecting approximately 30% of adults over the age of 60 and 62% of those over the age of 80 [1-3]. The unadjusted volume of all RTC repairs increased by 141% between 1996 and 2006 [1].  With the continually aging population, the incidence of tears and the number of surgical repairs is expected to rise commensurately [1,4-6].

Shoulder pain is initially assessed through in-office clinical evaluation with a detailed patient history and physical exam, including range of motion, muscular strength, and provocative tests utilized to narrow the clinical differential diagnosis [7,8]. Provocative testing of the RTC has been shown to be highly predictive of RTC pathology, including tears [9-11], but often advanced imaging is obtained to confirm the diagnosis.

While standard shoulder radiographs should be obtained for all patients presenting with shoulder pain, this imaging modality is insufficient for detecting RTC tears. In suspected RTC pathology cases, advanced imaging with magnetic resonance imaging (MRI) studies is preferred due to its high reliability in determining tear size, retraction, location, and atrophy [12]. Additionally, MRI can identify other coexisting soft-tissue injuries and is preferred for surgical planning [12,13].

Health insurance payers often mandate six weeks of conservative treatment with physical therapy (PT) within the last 12 weeks for patients who present with atraumatic shoulder pain prior to authorizing advanced imaging studies such as MRI [4,14-16]. Conservative pre-MRI protocol is designed to deter superfluous imaging orders; however, it may negatively impact patients for whom orthopedic specialists have determined that an MRI is warranted upon initial clinical evaluation [17-21]. Under the circumstance that examination by an orthopedic specialist raises high clinical suspicion for an RTC tear potentially warranting surgical repair, this insurance-mandated protocol may delay patient care, prolong patient discomfort, and increase risks associated with delayed surgical intervention.

Prior authorizations have increased in recent years, adding a significant burden to the medical system. Other specialties have looked at the burden, utility, and ethics of prior authorization [22-27]. Within orthopedic surgery, though, there are few studies reviewing its impact on clinical care. Kebaish et al. [27] retrospectively reviewed all spine MRIs ordered at a single institution and found that 85% required prior authorization, 10% required peer-to-peer review, and, of the 18% of MRI orders initially canceled or denied by this process, 77% were completed within three months. Only 85 of 2,480 orders were never done [27], raising concerns over the overall utility and ethics of this process. One of the main reasons for denial of the MRI in orthopedics is often the lack of six weeks of conservative care in the last 12 weeks. However, when a trained surgeon orders the imaging based on a clinical exam, is it reasonable for an insurance company to deny the request?

This study aimed to determine the positive yield of MRI-diagnosed full- and partial-thickness tears when ordered by an orthopedic specialist at the time of initial consultation for shoulder pain with concern for an RTC tear. The average cost of pre-MRI PT versus the cost of immediate MRI in these patients was also collected in the hope of identifying effective means of reducing healthcare costs.

Of note, this abstract was previously presented as a paper presentation/podium at the American Academy of Orthopaedic Surgeons on February 15, 2024, and as a poster at the International Congress on Shoulder and Elbow Surgery (2023) on September 5, 2023.

## Materials and methods

All patients 40 years or older who had an MRI ordered for suspicion of an atraumatic RTC tear on their first visit to a board-certified orthopedic surgeon at our institution were retrospectively identified. Institutional Review Board approval was obtained from our institution. Patients were identified by our medical informatics team utilizing our electronic medical records database and CPT code 73221 to identify patients who had an MRI ordered on their first visit with one of our orthopedic surgeons between January 1, 2015, to December 31, 2021. Medical informatics then cross-referenced each MRI ordered with the corresponding ICD 10 codes, M75.XX or S46.XX, to include patients who had an MRI ordered suspecting an RTC tear. Weakness documented on exam of RTC muscles concerning for tear warranted indication for the MRI ordered by the orthopedic specialist. Exclusion criteria included under 40 years of age; acute fracture or injury prior to the onset of pain, infection, or tumor in the shoulder of interest; prior history of ipsilateral shoulder surgery; previous ipsilateral upper extremity MRI; and/or incomplete MRI.

A total of 365 patients met the inclusion criteria. A retrospective review was performed to collect patient demographic data, including age, race, sex, body mass index (BMI), and insurance coverage. Insurance coverage was recorded as commercial health insurance, Medicare, Medicaid, and others (including self-pay and worker’s compensation). Clinical characteristics included ordering a physician to ensure there was no difference between surgeons; previous physical therapy of the ipsilateral shoulder; and previous corticosteroid injection (CSI) of the ipsilateral shoulder. At our institution, some primary care providers may order physical therapy for the patient or perform an injection prior to referral. These patients were included to impart an appropriate yield of tear accuracy based on clinical exam by an orthopedic surgeon. An independent cohort analysis was run on this subset of patients to ensure that this did not influence the data. Some insurance providers require physical therapy to be completed within the 12 weeks prior to ordering the MRI and will not accept physical therapy done more remotely for the same pain [16].

The primary outcome was the presence or absence of partial- or full-thickness RTC tear as documented by the MRI report and/or surgical operative notes when available. The secondary outcome was the subsequent surgical treatment of the tear following the MRI. Costs of upper extremity MRI without contrast and six weeks of PT sessions were derived from the publicly available financial pricing and transparency database provided by our institution.

Descriptive statistics and percentages were calculated to compare MRI outcomes and subsequent surgery rates. Statistical differences in demographic and clinical characteristics were analyzed with the Independent T-test and ANOVA for continuous parametric data (age, BMI) and chi-square test for categorical variables (race, sex, ordering physician, previous PT/injection, insurance coverage, and subsequent surgery). P-value <0.05 was utilized as a threshold for statistical significance.

## Results

Demographic analysis found no significant difference in sex, race, BMI, insurance coverage, ordering physician, or previous PT and/or CSI of the ipsilateral shoulder between patients with full, partial, or no tear present. The average age of patients with full-thickness, partial-thickness, and no tears differed significantly, with full-thickness tear patients being the oldest (61.12 ± 8.42, 58.46 ± 8.79, and 55.85 ± 8.86 years for full, partial, and no tears, respectively; p<0.001; Table 1). In patients with full-thickness tears, 70/157 (44.6%) had a prior history of PT and/or CSI, while 52/90 (57.8%) patients with partial-thickness tears, and 57/118 (48.3%) patients with no tears had a history of PT and/or CSI (Table 1).

**Table 1:** Patient Demographics Body Mass Index (BMI), Corticosteroid Injection (CSI), Full Thickness (FT), Physical Therapy (PT) F statistics performed via the one-way ANOVA; Y statistics performed via the chi-square test; *Statistical significance (p<0.05)

	No Tear (n=118)	Partial Tear (n=90)	FT Tear (n=157)	P-value
Age, years	55.85± 8.86	58.46±8.79	61.12± 8.42	<0.001*F
Race, n (%)				0.362Y
White	75 (64.1)	66 (73.3)	89 (56.7)	
Black	21 (17.9)	13 (14.4)	37 (23.6)	
Hispanic	11 (9.4)	5 (5.6)	19 (12.1)	
Asian	5 (4.3)	2 (2.2)	4 (2.5)	
Other	5 (4.3)	4 (4.4)	8 (5.1)	
Sex, n (%)				0.236Y
Male	56 (47.5)	45 (50.0)	90 (57.3)	
Female	62 (52.5)	45 (50.0)	67 (42.7)	
BMI, kg/m^2^	29.86±5.69	30.04±6.16	30.60±5.60	0.548F
Ordering physician, n (%)				0.083Y
1	51 (43.2)	30 (33.3)	39 (24.8)	
2	11 (9.3)	10 (11.1)	15 (9.6)	
3	8 (6.8)	6 (6.7)	13 (8.3)	
4	48 (40.7)	44 (48.9)	90 (57.3)	
Insurance, n (%)				0.226Y
Private	51 (43.2)	27 (30.0)	51 (32.5)	
Medicare	25 (21.2)	17 (18.9)	33 (21.0)	
Medicaid	38 (32.2)	39 (43.3)	66 (42.0)	
Other Insurance	4 (3.4)	7 (7.8)	7 (4.5)	
Previous shoulder PT and/or CSI, n (%)	57 (48.3)	52 (57.8)	70 (44.6)	0.134Y
No history of shoulder PT and/or CSI, n (%)	61 (51.7)	38 (42.2)	87 (55.4)	

Of the 365 patients included in the study, 157 (43.0%) had a full-thickness tear, 90 (24.7%) had a partial-thickness tear, and 118 (32.3%) had no tear (Table 2). Of the 186 patients who had no history of physical therapy and/or CSI of the ipsilateral shoulder, 125/186 (67.2%) had a positive MRI finding of a full-thickness (n=87) or partial-thickness (n=38) tear, while 61/186 (32.8%) were negative for an RTC tear (Table 2). In patients with a documented history of conservative treatment with a CSI or physical therapy (n=179), 122/179 (68.2%) demonstrated a positive MRI finding of a full-thickness (n=70) or partial-thickness (n=52) tear (Table 2).

**Table 2:** MRI Results Corticosteroid Injection (CSI), Full Thickness (FT), Magnetic Resonance Imaging (MRI), Physical Therapy (PT)

	Total Patients (n=365)	Previous Shoulder PT or CSI (n=179)	No Previous PT or CSI (n=186)
MRI FT Tear, n (%)	157 (43.0)	70 (39.1)	87 (46.8)
MRI Partial Tear, n (%)	90 (24.7)	52 (29.1)	38 (20.4)
MRI No Tear, n (%)	118 (32.3)	57 (31.8)	61 (32.8)

Patients with full-thickness tears (n=157) were further stratified by those who proceeded to surgery. Upon identification of a full-thickness tear, surgery was offered, and the election to undergo repair was at the discretion of the patient. A total of 88/157 (56.1%) of patients with a positive finding of a full-thickness tear underwent surgical repair. Of the 88 patients who had a full-thickness tear and underwent surgery, 35/88 had a history of previous PT and/or CSI, and 53/88 had no history (Table 3). Univariate analysis found no statistically significant differences in age, race, BMI, sex, ordering physician, insurance coverage, or history of physical therapy/steroid injection between patients with full-thickness tears who elected to undergo surgical repair and those who did not (Table 3).

**Table 3:** Univariate Analysis of Surgical Outcomes for Full-Thickness Tears Body Mass Index (BMI), Corticosteroid Injection (CSI), Full Thickness (FT), Magnetic Resonance Imaging (MRI), Physical Therapy (PT) F statistics performed via the independent T-test; Y statistics performed via the chi-square test

	FT-Surgery (n=88)	FT-No Surgery (n=69)	P value
Age, years	60.98±8.75	61.3±8.05	0.810F
Race, n (%)			0.575Y
White	48 (54.5)	41 (59.4)	
Black	19 (21.6)	18 (26.1)	
Hispanic	13 (14.8)	6 (8.7)	
Asian	2 (2.3)	2 (2.9)	
Other	6 (6.8)	2 (2.9)	
Sex, n (%)			0.885Y
Male	56 (56.8)	40 (58.0)	
Female	38 (43.2)	29 (42.0)	
BMI, kg/m^2^	30.75±5.31	30.39±5.99	0.691F
Ordering physician, n (%)			0.308Y
1	17 (19.3)	22 (31.9)	
2	8 (9.1)	87 (10.1)	
3	8 (9.1)	5 (7.2)	
4	55 (62.5)	35 (50.7)	
Insurance, n (%)			0.487Y
Private	29 (33.0)	22 (31.9)	
Medicare	15 (17.0)	18 (26.1)	
Medicaid	39 (44.3)	27 (39.1)	
Other Insurance	5 (5.7)	2 (3.9)	
Previous shoulder PT and/or CSI, n (%)	35 (39.8)	35 (50.7)	0.171Y
No history of shoulder PT and/or CSI, n (%)	53 (60.2)	34 (49.3)	

Reviewing charges for MRI and PT at our institution, we found that the cost of an upper extremity MRI without contrast averages $2,268. A single PT session costs an average of $194, with an estimated two sessions per week for six weeks totaling $2,328.

## Discussion

Insurance payers often require a minimum of a six-week period of conservative treatment within the last 12 weeks for patients who present with shoulder pain prior to authorizing advanced imaging studies [3,15,17]. The goal of this study was to determine the incidence of RTC tears in patients who had an MRI ordered upon initial consultation with an orthopedic specialist due to concern for RTC tears. Over 67% of MRI orders analyzed yielded a positive finding of either a partial or full-thickness RTC tear. The positive yield of MRI imaging remained at 67.2% in the absence of a history of conservative treatment, validating a specialist's clinical suspicion of tear and indication for MRI.

Physical therapy has been shown to be very effective in eliminating shoulder pain in certain conditions; however, there are patients whose history and physical exam prompt concern for pathology that may not be appropriately treated with PT. Moosmayer et al. [28,29] compared the management of small- and medium-sized cuff tears at one-year and 10-year follow-ups and demonstrated that primary tendon repair results in superior outcomes when compared to nonoperative management. In patients with full-thickness RTC tears, it has similarly been demonstrated that patients who elected to undergo surgical repair reported greater improvement in functional outcomes and reduced pain when compared to those who were treated conservatively [30]. Delaying RTC surgery ≥1 year has also been shown to result in higher retear rates after RTC repair [31]. Of the patients with an identified full-thickness tear by MRI order on initial consultation for chronic shoulder pain in our study, 56.1% underwent surgical tendon repair. Thus, in these patients with tears that warrant surgical intervention, six weeks of required PT could have delayed treatment whilst adding to healthcare expenditure. It is important to acknowledge, however, that the goal of this study is not to discredit the value of PT, but instead seek to emphasize the importance of physician-directed image ordering without insurance or third-party interference.

At our institution, the cost of an upper extremity MRI without contrast averages $2,268. A single PT session costs an average of $194, with an estimated two sessions per week for six weeks totaling $2,328, thus doubling the healthcare cost if patients are likely to proceed with surgery once diagnosed with a tear. Malik et al. [18] noted that an important factor driving the high utilization of healthcare resources before arthroscopic RTC repair for atraumatic tears is the insurance-mandated trial of nonoperative interventions before authorizing a surgical procedure [18]. It is estimated that 40%-90% of one-year per-patient average reimbursements are attributed to radiographs, physical therapy, opioids, steroid injections, and office visits within the three months prior to arthroscopic RTC repair surgery [32]. Judicious use of physical therapy prior to MRI authorization may offer an effective way to reduce healthcare costs and reserve PT sessions for post-operative rehabilitation in patients who are identified by a board-certified orthopedic specialist as having a high risk of RTC tear during the index consultation.

Prior authorization requirements are a significant burden to practitioners, with an estimated time to the dollar cost of $78,913 and $66,954 spent annually per medical specialist and surgeon, respectively, and overall estimated national time cost to practices of interactions with insurance plans of $23 billion to $31 billion each year [20]. Streamlining MRI orders from specialists would further reduce lost productivity time and unaccounted costs while decreasing risks associated with delayed surgical intervention. In turn, this can contribute to decreased healthcare costs, improved patient care, and increased patient satisfaction.

The present study has several limitations. First, the design is a retrospective cohort study and therefore is prone to inherent biases of retrospectively collected data. Second, the patient sample included in this study was collected from a single institution. Thus, the external validity and reproducibility of our findings may be limited. Third, the length or type of previous PT patients underwent was not assessed. Patients included in the study cohort had documentation of conservative PT, but there was no description of the sessions or weeks completed. The type of conservative management was also not documented in patient charts, which can include non-standardized combinations of physical exercises, manual therapy, and loading [33]. Therefore, the length and type of PT could affect the average cost, as well as a functional benefit due to conservative management prior to RTC surgery. Additionally, we did not control for concomitant medical pathologies or medications, which may influence the risk of RTC tear. Future research delineating postoperative outcomes between patients who received preauthorization for MRI and those who underwent mandated six-week conservative therapy would provide further clarification on this topic.

## Conclusions

This study demonstrates that orthopedic specialists have a high yield rate when identifying RTC tears by MRI based on history and physical exam upon initial consultation. In patients who present with high clinical suspicion for RTC tear, mandated six weeks of PT by insurance companies delays early MRI diagnosis and thus potential intervention. Additional pre-MRI PT sessions and disputes with insurance companies over prior authorization may also contribute to increased healthcare expenditures. Ultimately, indication for timely imaging versus conservative therapy may be better advocated by a trained orthopedic specialist rather than insurance companies.
